# “A dual-applanation physical model to improve accuracy in goldmann tonometry by accounting for corneal biomechanics”

**DOI:** 10.3389/fbioe.2026.1757214

**Published:** 2026-04-29

**Authors:** Gaetano Barbaro, Ciro Caruso, Mario Troisi, Ciro Costagliola

**Affiliations:** 1 Istitute Refractive Ophthalmology Surgery Napoli, Naples, Italy; 2 Clinica Oculistica, Pellegrini Hospital, ASL Napoli 1, Naples, Italy; 3 UOC di Oculistica, Ruggi d’Aragona Hospital, Salerno, Italy; 4 Clinica Oculistica, Università degli Studi di Napoli Federico II, Naples, Italy

**Keywords:** corneal biomechanics, goldmann tonometry, intraocular pressure, IOP error correction, mathematical model

## Abstract

**Purpose:**

We developed and analytically evaluated a novel physical-mathematical model aimed at quantifying and minimizing the systematic error (*ERR*) between true intraocular pressure (*IOPT*) and that measured by Goldmann applanation tonometry (*IOPG*), by incorporating patient-specific corneal biomechanical properties.

**Methods:**

We constructed a model based on physical force balance, simulating the applanation of the cornea as a deformable elastic shell influenced by intraocular pressure and external forces. Four distinct forces were mathematically defined and computed: F1 – intraocular pressure acting on the posterior corneal surface; *F2* – elastic resistance of the corneal tissue to flattening; *F3* – adhesive force due to tear film surface tension; *F4* – the net force applied by the examiner and recorded by the *GAT* device. The model integrates key anatomical and biomechanical parameterscentral corneal thickness, mean apical curvature radius, and corneal elastic modulus—to quantify *ERR* as a function of individual variability. These parameters are derived from two sequential applanation measurements (*IOPG*_*0*_ *and IOPG*_*1*_), acquired through a custom-designed dual-zone applanation prism.

**Results:**

Simulations demonstrated that *ERR* increases proportionally with central corneal thickness and elastic modulus, and decreases with increasing corneal curvature radius. For standard corneal values (thickness: *0.536 mm*; curvature: *7.15 mm*; elasticity: *0.16 MPa*), the model closely approximates *IOPT*. Deviations from these reference values result in systematic over- or underestimation of *IOPG*.

**Conclusion:**

This analytical model provides a transparent, biomechanically grounded method for correcting *GAT* measurements and estimating true *IOP* with improved precision. It highlights the critical influence of patient-specific corneal properties and lays the foundation for personalized tonometry in clinical practice.

## Highlights

- Due to the heterogeneity in the anatomical characteristics of the cornea, the Goldmann applanation tonometry pressure (*IOPG*) is not always the “TRUE” *IOP* (*IOPT*).
- We developed a physical-mathematical model that assesses and computes each of the physical forces involved in the Goldmann applanation tonometry (*GAT*), according to the variability of the physical features of each applanated cornea.
- We have estimated the *GAT* error, which is the difference between our calculation and the *GAT* calculation, and how this error increases with different corneal factors including thickness, curvature, and elasticity.

## Introduction

Intraocular pressure (*IOP*) remains a key clinical biomarker for the diagnosis, monitoring, and management of glaucoma and other optic neuropathies. Among various techniques, Goldmann applanation tonometry (*GAT* introduced in 1957), has long been considered the gold standard for *IOP* measurement due to its relative simplicity and clinical reliability (Goldmann and Schmidt, 1957). *GAT* operates by flattening a predefined central corneal area (*3.06 mm* in diameter), under the assumption—based on Goldmann and Schmidt’s original experiments—that the external applanation force directly correlates with true intraocular pressure (*IOPT*) (Goldmann and Schmidt, 1957). However, subsequent studies have shown that this assumption does not always hold true, particularly due to individual anatomical and biomechanical variability in the cornea (Chihara, 2008; Whitacre et al., 1993). Despite these efforts, a universally accepted correction methodology for *GAT* does not yet exist, and the clinically measured *IOPG* often deviates from the true *IOPT* in a non-linear and individualized manner (Chihara, 2008). Central corneal thickness (*CCT*), curvature, and the corneal elastic modulus are all known to significantly affect the accuracy of *GAT* measurements. As demonstrated by Whitacre and Stein, *GAT* tends to overestimate *IOP* in eyes with thicker corneas and underestimate it in thinner corneas, thus introducing clinically relevant bias (Whitacre and Stein, 1993). Several attempts have been made to address this issue using mathematical models and empirical correction formulas. For instance, Orssengo and Pye proposed a biomechanical model to estimate both true IOP and the corneal modulus of elasticity *in vivo*, yet the complexity of their equations hindered widespread clinical adoption (Orssengo and Pye, 1999). More recently, finite element simulations have been used to evaluate the corneal response to applanation. Liu and Roberts found that corneal deformation under pressure is highly dependent on the biomechanical properties of the tissue (Liu and Roberts, 2005), and Elsheikh et al. demonstrated that corneal stiffness and nonlinearity contribute significantly to the *GAT* error (Elsheikh et al., 2006). Other studies have investigated the impact of tear film surface tension on *GAT* readings. Tiffany et al. quantified this parameter in both normal and dry eye conditions, highlighting its potential to introduce an additional force *F3* that affects the tonometric measurement (Tiffany et al., 1989). This study introduces a novel physical-mathematical model that analytically reconstructs the forces involved in *GAT*, specifically accounting for biomechanical variability in each applanated cornea. The model decomposes the total applanation force (*F4*) into four components: (*F1*), the true intraocular pressure acting against the cornea; (*F2*), the elastic resistance of the cornea; (*F3*), the adhesive force due to tear film surface tension and (*F4*), the net external force applied by the examiner, which is read by the Goldmann tonometer. By integrating corneal thickness, curvature, and modulus of elasticity into force-based equations, the model allows for quantification of the measurement error (*ERR = IOPG - IOPT*) and provides a method to estimate *IOPT* more accurately. While previous analytical and finite-element models have described corneal deformation under applanation, the novelty of the present study lies in deriving a closed-form analytical solution that directly links two sequential applanation measurements to true intraocular pressure, corneal elastic modulus, and systematic Goldmann error within a unified physical framework.

## Materials and methods

When the cornea is flattened during Goldmann Applanation Tonometry (*GAT*), the tonometer reading represents the resultant of several forces acting on the applanated surface. Theoretical modeling identifies at least four contributing forces:

- (F1): the force exerted by the true intraocular pressure (*IOPT*).
- (F2): the elastic reaction force of the applanated portion of the cornea.
- (F3): an attractive force generated by the tear film, which tends to retain the cone against the cornea, thereby opposing the elastic recoil of the deformed tissue.
- (F4): the thrust applied by the examiner through the tonometer rod. This is the force recorded by the Goldmann tonometer, and can be expressed as: (*F4 = F1 + F2-F3*).

### Forces (F1 and F2)

**FIGURE 1:**
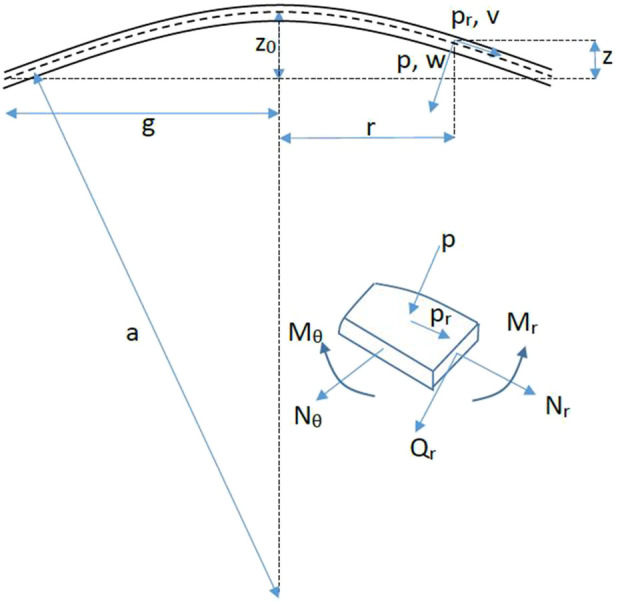
Schematic view of a cornea flattened during *GAT*: physical parameters of the radial and tangential displacement.

To accurately compute (*F1*) and (*F2*), we analyze the elastic deformation of a spherical shell characterized by a uniform thickness significantly smaller than both its diameter and radius of curvature, and assumed to be rigidly fixed at its base. The corneal shell, filled with aqueous humor exerting a pressure *IOPT*, is defined by an internal radius *RINT*, an external radius *REXT*, and a modulus of elasticity *E*, which describes its material stiffness. (See Appendix A). To account for differences between the inner and outer surfaces of the cornea, we define an intermediate surface with radius *a*, calculated as the arithmetic mean of *REXT* and *RINT*. Given that corneal thickness is much smaller than the curvature radius of this intermediate surface, we assume that the forces acting at the boundaries are equivalent to those acting along the intermediate surface. Upon applanation by the *GAT* cone, the intermediate surface (with radius *g*) is flattened, as shown in (Figure 1). This necessitates analysis of both radial and tangential displacements. The radial displacement *w* of the intermediate surface is defined as follows:

$$\frac{w}{a}=1-\sqrt{\frac{a^{2}-g^{2}}{a^{2}-r^{2}}}$$

The tangential displacement (*v = 0*) is neglected, based on the assumption that the cornea deforms primarily in the radial direction toward its center. Tangential displacement was neglected (*v = 0*) as a first-order approximation. Dimensional estimates indicate that in-plane strain energy contributions remain small compared to bending energy within the applanation region under physiological loading conditions. (See Supplementary Appendix B). A zero contact angle was assumed to represent complete wetting conditions typically achieved during fluorescein-assisted applanation. While tear film properties may vary, this approximation isolates the primary Laplace contribution without introducing additional geometric complexity.

### (F3) force

**FIGURE 2:**
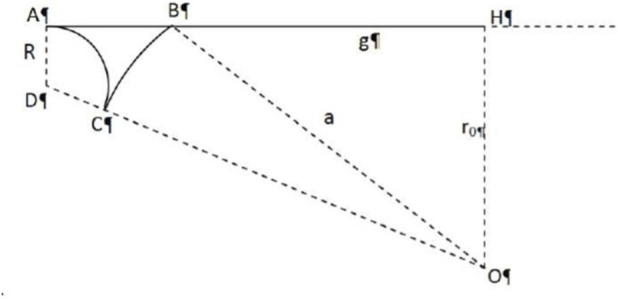
Geometrical representation of the lacrimal meniscus (*ABC*: tear meniscus formed during the applanation; *BC*: tear film; *AH*: smoothing surface of the cone). Schematic representation of the tear meniscus that holds the tonometer head against the cornea (*ABC*: tear meniscus formed during the applanation; *BC*: tear film; AH: smoothing surface of the cone).

As shown in (Figure 2), during applanation a tear meniscus (*ACB*) forms, linking the flattened corneal surface (*AH*) with the tear film remaining on the unflattened portion (*BC*). We assume that: the contact angle between the meniscus and the cone surface is zero, indicating perfect wetting; and the meniscus also forms a zero-degree contact angle with the corneal surface. This assumption leads to an overestimation of the force (*F3*), because in general the contact angle between the meniscus and the cone surface is not zero.

### Modified applanation prism design

To enable simultaneous acquisition of both *IOPG*_*0*_ and *IOPG*_*1*_ values, a modified applanation cone was developed. The device incorporates a dual optical prism system that generates two concentric applanation zones: the standard *3.06 mm* diameter zone used in Goldmann tonometry, and an additional outer zone with a diameter of *3.60 mm*. Both zones are optically aligned and visible through the same slit-lamp biomicroscope.

During the measurement, the clinician observes two pairs of fluorescein patterns: two inner semicircles corresponding to *IOPG*_*0*_ *,* and two outer arcs corresponding to *IOPG*_*1*_. This dual-view system allows sequential pressure readings from concentric corneal areas without repositioning the instrument. These two independent values, acquired under standardized conditions, are then inserted into the mathematical model to compute both the true intraocular pressure (*IOPT*) and the corneal Young’s modulus (*E*).

**FIGURE 3:**
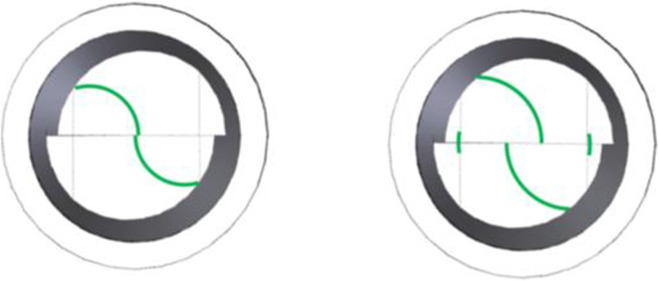
Visual appearance through the Goldmann tonometer prism (left) and through the modified dual-measurement prism (right). Left image: Standard applanation interface with a *3.06 mm* diameter contact zone, showing the typical alignment of two semicircular fluorescein rings used to determine *IOPG*_*0*_ (Goldmann-derived intraocular pressure). Right image: Appearance through the newly developed prism, which retains the standard central measurement (*IOPG*_*0*_) while simultaneously revealing two additional peripheral arcs corresponding to an outer concentric applanated zone of *3.60 mm* diameter. This configuration enables a second sequential pressure measurement (*IOPG*_*1*_). Both *IOPG*_*0*_ and *IOPG*_*1*_ are integrated into the mathematical model to estimate the true intraocular pressure (*IOPT*) and, uniquely, to derive the *in vivo* corneal Young’s modulus (*E*), based on patient-specific biomechanical parameters.

The visual result of this modified optical design is illustrated in (Figure 3), which shows the comparison between the classic Goldmann view and the enhanced dual-applanation configuration.

### Force (F1)

$The\;mean\;pressure\;\frac{F1}{\pi \;g^{2}}$ acting on the smoothed intermediate surface of the corneal cap, due to intraocular pressure, is equivalent to the intraocular pressure itself. Consequently:

$$F1={IOP}_{T}\cdot \pi \;\cdot g^{2}$$

### Force (F2)

**FIGURE 4:**
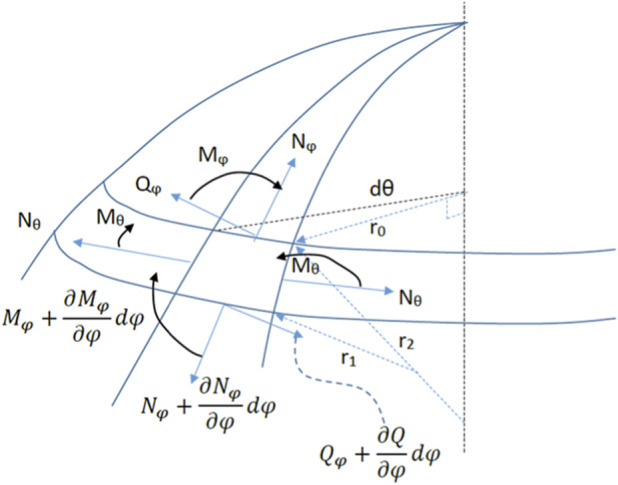
Geometric representation of direct tension, shear stress, radius of curvature, and bending moments along the longitudinal and latitudinal polar coordinates and in the radial direction.

To evaluate the elastic deformation of a homogeneous spherical cap with uniform thickness h, external radius *REXT*, and internal radius *RINT*, one must consider the balance of forces and bending moments acting on an infinitesimal element of the cap, as illustrated in Figure 4. This leads to the derivation of the elastic deformation equations (Timoshenko and Goodier, 1970). The deformation regime considered corresponds to small central deflections under localized loading. In this range, bending stiffness contributes significantly to the overall mechanical response, justifying the use of bending-dominated thin shell theory. Membrane-dominated behavior becomes relevant under large global deformations, which are outside the scope of Goldmann applanation tonometry (See Supplementary Appendix A). The limbus was modeled as a clamped boundary condition. While physiological fixation is not perfectly rigid, this assumption provides a conservative and analytically tractable approximation consistent with classical thin-shell formulations.

**FIGURE 5:**
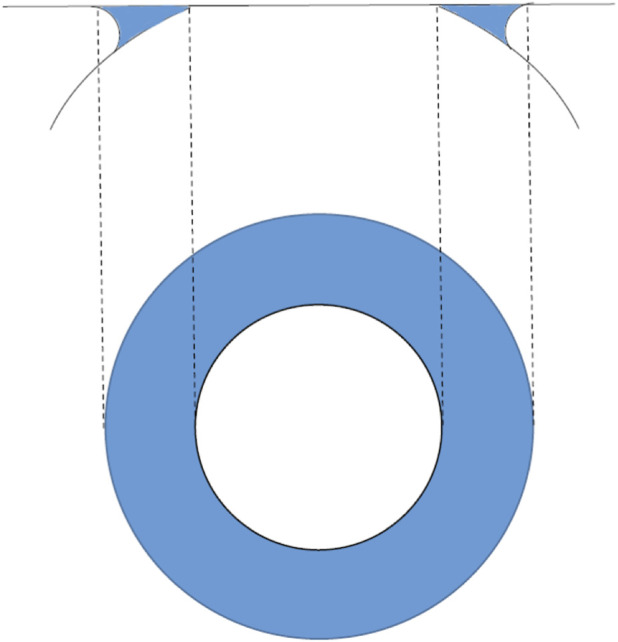
the tear meniscus, around the perimeter of the applanated corneal surface, that attracts the tonometer cone against the cornea.

Force (*F3*) In the present model, the tear film surface tension was assumed to be *γ ≈ 0.043 N/m*, consistent with physiological values reported in the literature. Substituting this value into the Laplace-derived expression (*F*_*3*_ *= 2γ/r*), and using the standard Goldmann applanation radius (*r = 1.53 mm*), yields an equivalent pressure correction of approximately *4.0 mmHg*. The constant term adopted in the analytical formulation therefore directly reflects this physiological assumption. Figure 5 illustrates the formation of a tear meniscus surrounding the perimeter of the applanated corneal surface, which exerts a holding force on the tonometer head (Yokoi et al., 2003). According to Laplace’s law, the pressure difference *Δp* between the two regions separated by the air-liquid interface is proportional to the sum of the principal radii of curvature (See Supplementary Appendix B).

Thus, the force (*F3*) does not depend in a first approximation on the thickness of the tear film. This can be explained because the force (*F3*) is obtained by multiplying the force acting per unit of wet surface, which is inversely proportional to the radius of curvature *R* of the meniscus, with the area of the annular wetted surface, which is directly proportional to the distance (*AB*) (this distance is directly proportional to the radius of curvature *R* of the meniscus). Consequently, the force (*F3*) in a first approximation can be considered independent from the radius of curvature of the meniscus and therefore of the thickness of the tear film, as long as the distance (*AB*) is small compared to the radius of the smoothing surface (*BH*). Accurate measurement of the surface tension *λ* of the tear film is therefore essential for a correct evaluation of the force (*F3*). If the tear film surface tension can be approximated to the distilled water surface tension, then the difference in mean pressure caused by the tear film on to the smoothed corneal surface can be estimated as follows:

$$2\cdot \frac{a+a_{0}}{{\bar{BH}}^{2}}\cdot \lambda_{H2O}≅6.6\;mmHg$$

The force (*F3*), when the applanation radius *g* is equal to *1.53 mm*, is therefore approximately equal to:

$$F3≅2\cdot \frac{a+a_{0}}{{\bar{BH}}^{2}}\cdot \lambda_{H2O}\cdot \pi \cdot {\bar{BH}}^{2}=2\cdot \left(a+\sqrt{a^{2}-g^{2}}\right)\cdot \lambda_{H2O}\cdot \pi ≅6.5\;mN$$

If we consider defined surface tension values, as calculated in other studies (Yokoi et al., 2003) for normal and dry eye, the force (*F3*) assumes the following values:

Normal eye:

$$F3≅2\cdot \frac{a+a_{0}}{{\bar{BH}}^{2}}\cdot \lambda_{normal\;}\cdot \pi \cdot {\bar{BH}}^{2}=2\cdot \left(a+\sqrt{a^{2}-g^{2}}\right)\cdot \lambda_{normal\;}\cdot \pi ≅3.9\;mN$$

for an average pressure difference (referred to the smoothed corneal surface) caused by the tear film equal to *4.0 mmHg*.

Dry eye:

$$F3≅2\cdot \frac{a+a_{0}}{{\bar{BH}}^{2}}\cdot \lambda_{dry}\cdot \pi \cdot {\bar{BH}}^{2}=2\cdot \left(a+\sqrt{a^{2}-g^{2}}\right)\cdot \lambda_{dry}\cdot \pi ≅4.5\;mN$$

for an average pressure difference (referred to the smoothed corneal surface) caused by the tear film equal to *4.6 mmHg*.

## Results

The present model yields two fundamental equations for the quantification of intraocular pressure corneal elasticity and error in *IOP* measurement (*ERR*). These results are based on the biomechanical and physical modeling of the cornea as a spherical shell subject to applanation forces.

1. Estimation of true intraocular pressure (*IOPT*)

The true intraocular pressure (*IOPT*) can be calculated from the balance of physical forces involved in applanation tonometry, using the following expression (Equation 1):

$$IOPT≅{IOPG}_{0}+\frac{{IOPG}_{0}\cdot g_{1}^{2}-{IOPG}_{1}\cdot g_{0}^{2}}{\frac{g_{1}^{4}}{g_{0}^{2}}-g_{1}^{2}}+4.0mmHg\cdot \frac{g_{1}^{2}+g_{0}^{2}}{g_{1}^{2}}$$ (1)

2. Estimation of corneal modulus of elasticity (E)

The model also enables the *in vivo* estimation of the corneal modulus of elasticity. The following equation was derived from the force balance at equilibrium during applanation (Equation 2):

$$E≅\frac{a^{3}}{h}\cdot \frac{{IOPG}_{1}\cdot \left(1-\frac{g_{0}^{2}}{g_{1}^{2}}\right)-{IOPG}_{0}\cdot \left(\frac{g_{1}^{2}}{g_{0}^{2}}+1\right)+4.0mmHg\cdot \frac{g_{1}^{2}+g_{0}^{2}}{g_{1}^{2}}\cdot \left(\frac{g_{1}^{2}}{g_{0}^{2}}-1\right)}{\frac{g_{1}^{4}}{g_{0}^{2}}-g_{0}^{2}}$$ (2)

3. Estimation of error in *IOP* measurement (*ERR*)

The error (*ERR*), caused by the approximation of the true intraocular pressure *IOPT* with the pressure measured with *GAT* is equal to Equation 3:

$$ERR=\frac{{E\cdot h\cdot g}^{2}}{2\left(1-v\right)a^{3}}-4.0$$ (3)

The equations were evaluated across a range of input values for CCT (450–650 μm), curvature radius (*6.5–8.0 mm*), and known surface tension coefficients of the tear film (*λ = 0.044–0.073 N/m*). Computed values of *IOPT* were found to deviate from *IOPG* by *0.5 to 7 mmHg*, depending on the combination of biometric parameters used. Correspondingly, the calculated corneal modulus of elasticity ranged between 0.10 and *0.35 MPa* under physiologic conditions. All simulations were performed using nominal values consistent with published literature and clinical biometric distributions.

## Discussion

Intraocular pressure (*IOP*) remains a cornerstone in the diagnosis and management of glaucoma. While direct manometry provides accurate measurements, its invasive nature restricts its use to experimental settings. As a result, indirect methods such as tonometry—and in particular, Goldmann applanation tonometry (*GAT*)—have become the clinical standard^1^. *GAT* relies on the assumption that flattening a *3.06 mm* corneal area yields a force equal to the true *IOP* (*IOPT*). However, numerous studies have demonstrated that this assumption fails in the presence of interindividual variability in corneal properties such as thickness, curvature, and biomechanical resistance (Chihara, 2008; Whitacre et al., 1993). However, the model assumed a constant and homogeneous modulus of elasticity (*E*) throughout the shell, independent of the acting forces a simplification that contradicts biological reality. Liu and Roberts (2005) further demonstrated that variations in corneal stiffness, thickness, and curvature significantly affect *GAT* measurements (Liu and Roberts, 2005). Schwartz et al. (1966) conducted a rigorous theoretical study of corneal deformation during tonometry using elastic shell theory. Despite its mathematical robustness, the model’s complexity rendered it clinically inaccessible (Schwartz et al., 1966). Attempts by Orssengo and Pye to derive true *IOP* and corneal elasticity *in vivo* resulted in equations difficult to implement in practice (Orssengo and Pye, 1999). Finite element models, such as those by Elsheikh et al. (2006), introduced improved realism, but are still limited by computational burden and lack of integration with clinical devices (Elsheikh et al., 2006). The unresolved problem in Goldmann tonometry is the simultaneous determination of true intraocular pressure and corneal stiffness from applanation data without empirical correction coefficients. The dual-applanation model addresses this inverse problem analytically, enabling extraction of both *IOPT* and *E* from two independent force-balance equations derived from first principles. Existing biomechanical correction devices (ORA, Corvis ST, CID, CATS) rely on empirically derived indices or device-specific calibration models. In contrast, the present approach provides a purely physics-based analytical correction grounded in measurable geometric and mechanical parameters. A major limitation shared by many of these models is the oversimplification of the cornea as a membrane with negligible thickness. This leads to overestimations of the force (*F1*), because it ignores the smaller internal applanation surface relative to the external one. Furthermore, these approaches often neglect the biomechanical effect of the tear film, which exerts an adhesive force (*F3*) that counteracts elastic recoil during measurement (Tiffany et al., 1989; Yokoi et al., 2003). To address these gaps, our model reconstructs the *GAT* process through the explicit calculation of four physical forces: (*F1* = intraocular thrust), (*F2* = corneal elastic resistance), (*F3* = tear film adhesion), and (*F4* = net force applied by the examiner). We model the portion of the cornea that is flattened during Goldman tonometry as a spherical cap of uniform thickness, incorporating both the internal and external surfaces. The analytical formulation is rooted in the elastic shell theory of Timoshenko, which allows for the integration of bending moments, radial and tangential displacements, and material-specific elastic behavior (Timoshenko and Goodier, 1970). Importantly, we incorporate the Laplace-derived force due to tear film surface tension (*F3*), providing a more comprehensive understanding of the cornea–tonometer interaction. This force, often estimated around *4.0 mmHg* under physiological tear conditions, plays a non-negligible role in applanation mechanics, particularly in dry eye or altered tear film composition (Timoshenko and Goodier, 1970; Yokoi et al., 2003). Variability in fluorescein distribution and minor alignment errors during applanation may alter the effective contact area and influence the measured force balance. These factors represent known sources of variability in Goldmann tonometry and are not unique to the dual-applanation configuration. The assumption of negligible contact angle approximates clinical Goldmann tonometry under fluorescein instillation, where tear meniscus wetting approaches complete spreading. While tear film properties may vary in dry eye conditions, the analytical formulation demonstrates that the capillary force term is first-order independent of tear film thickness. Nevertheless, spatial and temporal variability in surface tension represents a potential source of measurement uncertainty that warrants future investigation.

Our simulations show that the error (*ERR*) in *IOP* measurement correlates with biomechanical parameters: *ERR* increases linearly with both central corneal thickness and elastic modulus, and decreases with increasing curvature radius. These insights validate clinical observations and offer a mechanistic explanation for previously empirical finding (Chihara, 2008; Orssengo and Pye, 1999; Elsheikh et al., 2006).

In Goldmann original hypothesis, the cornea was considered a spherical membrane without a significant thickness. Unlike previous analytical or finite-element models that estimate corneal deformation numerically or introduce empirically calibrated correction factors, the present framework derives a closed-form analytical solution directly from force equilibrium principles. The innovation does not reside in remodeling corneal deformation *per se*, but in integrating a dual-applanation measurement strategy with a physics-based analytical formulation that simultaneously solves for true intraocular pressure and corneal elastic modulus without reliance on empirical fitting parameters. Although previous analytical models addressed estimation of true *IOP* and corneal stiffness, the present work introduces a dual-applanation implementation yielding a closed-form analytical solution derived directly from force-balance principles, without reliance on empirical correction factors. The model here proposed approximates the human cornea with the following limitations: in the present model, Goldmann applanation induces a highly localized deformation of the cornea, with an apex displacement (∼0.3 mm) that is negligible compared to the axial length of the eye (∼23 mm). Therefore, the influence of axial length on the mechanical response involved in applanation is expected to be minimal. However, we acknowledge that axial length may play a role in specific conditions (e.g., highly myopic eyes) and should be considered in future model extensions, the human cornea does not have the shape of a spherical cap; the corneal thickness is not uniform; the cornea is not made of homogeneous material, but it is made of different layers; although corneal tissue exhibits viscoelastic behavior, the deformation induced by Goldmann applanation tonometry is limited and quasi-static. Under these conditions, the elastic response is expected to dominate, while viscoelastic effects such as hysteresis are likely to play a secondary role. This assumption represents a simplification of the model and may influence its accuracy under dynamic conditions; the cornea is not rigidly fixed but is fixed to the eye through elastic tissues. Furthermore, the present model focuses on the local corneal response, as Goldmann applanation involves a confined central area with stress rapidly decreasing toward the periphery. Consequently, the contribution of global ocular rigidity, including scleral biomechanics, is expected to be limited under these conditions, although it may become relevant in cases involving larger-scale deformation. These limits appear negligible because the smoothed corneal surface is small compared to the total surface of the cornea, so: the corneal surface that will be touched by the smoothing surface can be approximated as a spherical cap; the corneal thickness of the applanated area typically varies by less than ten percent, so it can be considered practically constant; the ratio *E* between the smoothing force and the induced corneal deformation can be considered as an overall average modulus of elasticity of the multi-layer structure of the cornea; the portion of the cornea that undergoes some significant changes during applanation tonometry is the flattened surface and not the base of the cornea, which is thicker. Hence, the deformation induced in the cornea during the measurement is practically limited to the smoothed surface. Furthemore, sequential applanation is performed within the elastic deformation regime and within a short time interval insufficient to induce viscoelastic creep or structural alteration. The magnitude of deformation remains well below thresholds associated with corneal microstructural remodeling, supporting the assumption of reversible behavior during measurement.

*IOPT* allows retrospective correction of the Goldmann Applanation Tonometry (*GAT*) reading (*IOPG*) by accounting for patient-specific parameters such as central corneal thickness (*CCT*), curvature radius, and elastic modulus (*E*).

The model also enables the *in vivo* estimation of the corneal modulus of elasticity.

Equation 1 describes the mmHg reading of the *IOP* in *GAT*. Equation 2 was derived from the force balance at equilibrium during applanation.

It represents the first mathematical approach for calculating the Young’s modulus *E* of the human cornea non-invasively, using clinical data acquired during standard tonometric assessment.

**FIGURE 6:**
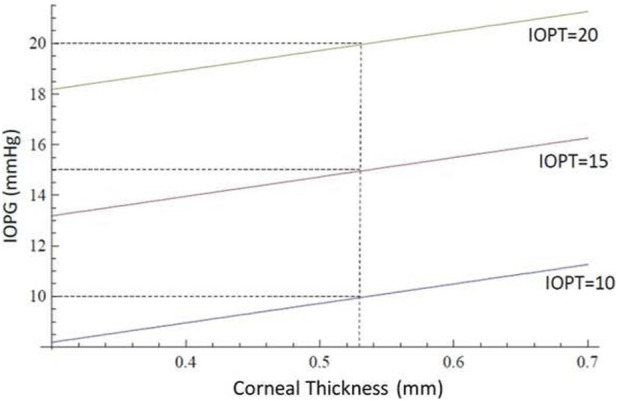
The abscissas and ordinates show a theoretical simulation illustrating the influence of corneal thickness on *GAT*. The *IOPG* measurement obtained with *GAT* coincides with the true *IOPT* only when the corneal thickness equals *0.536 mm*. For the same *IOPT* value, *IOPG* increases with increasing thickness. The data were calculated with fixed values of mean corneal curvature (*7.15 mm*) and modulus of elasticity (*0.16 MPa*).

**FIGURE 7:**
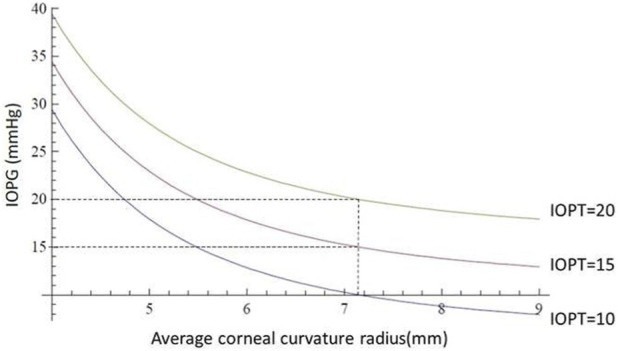
The abscissas and ordinates show a theoretical simulation illustrating the influence of the corneal apical mean radius of curvature on intraocular pressure measurement with the Goldmann tonometer. The *IOPG* measurement coincides with the true *IOPT* only when the average corneal apical curvature radius equals *7.15 mm*. For the same *IOPT* value, *IOPG* decreases as the radius of curvature increases. The data were calculated with fixed values of corneal thickness (*0.536 mm*) and modulus of elasticity (*0.16 MPa*).

**FIGURE 8:**
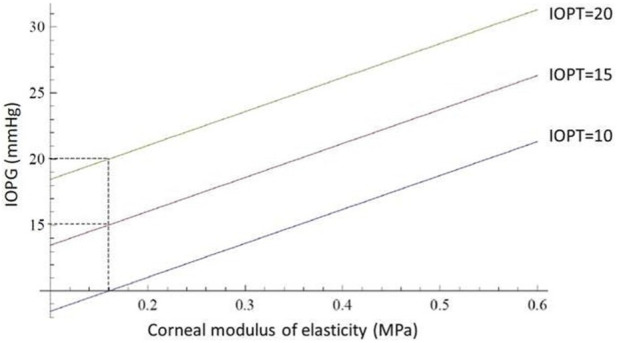
The abscissas and ordinates show a theoretical simulation illustrating the influence of the corneal modulus of elasticity on intraocular pressure measurement with the Goldmann tonometer. The *IOPG* measurement coincides with the true *IOPT* only when the corneal modulus of elasticity equals *0.16 MPa*. For the same *IOPT* value, *IOPG* increases as the modulus of elasticity increases. The data were calculated with fixed values of corneal thickness (*0.536 mm*) and mean apical radius of curvature (*7.15 mm*).

**FIGURE 9:**
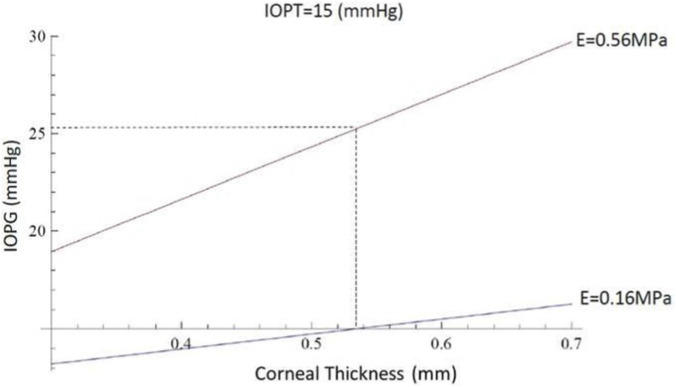
The abscissas and ordinates show how the influence of corneal thickness on IOP measurement depends on the biomechanical properties of the cornea. A higher modulus of elasticity corresponds to a steeper slope, while a lower modulus corresponds to a shallower slope.

**FIGURE 10:**
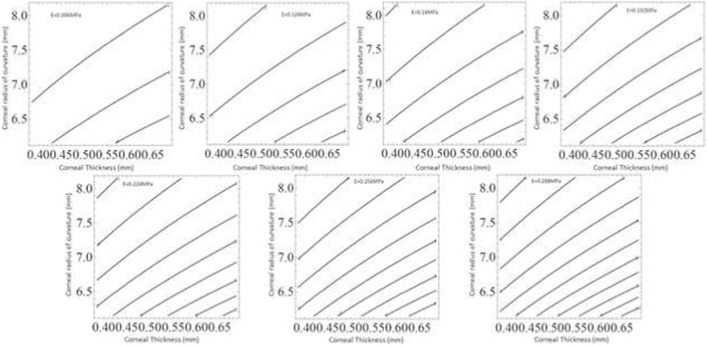
It shows the error *ERR* (measured in mmHg), which occurs in approximating the *IOPT* true intraocular pressure value with the measurement given by the Goldmann *IOPG* tonometer, related to the variation of the corneal thickness, of the corneal apical average radius of curvature and of the corneal modulus of elasticity (*E*). These figures can be used to correct the measurement made with the tonometer according to the patients modulus of elasticity.

The error (Equation 3) in the measurement of intraocular pressure with the Goldmann tonometer is therefore influenced by three parameters at the same time: it increases as the product between the corneal modulus of elasticity and the apical corneal thickness increases and decreases with the cube of the mean apical corneal curvature radius. Beyond its theoretical formulation, the proposed model delivers a practical mathematical framework for quantifying the measurement error (*ERR*) between *IOPG* and *IOPT*. Our equations allow for an explicit calculation of *ERR* as a function of three key corneal parameters: central corneal thickness (*CCT*), corneal radius of curvature, and the modulus of elasticity (*E*). The simulations (Figures 6–10) reveal that *ERR* increases almost linearly with *CCT* (Figure 6), assuming fixed values for curvature and elasticity. This aligns with established clinical findings that *GAT* tends to overestimate *IOP* in thicker corneas and underestimate it in thinner ones (Whitacre et al., 1993; Whitacre and Stein, 1993). However, our model quantifies this relationship explicitly and enables a direct correction based on measured *CCT*. Interestingly, the model also demonstrates that *ERR* increases polynomially as the radius of curvature increases (Figure 7), when thickness and elasticity are held constant. This result adds a new dimension to the interpretation of *GAT* inaccuracies, as corneal curvature has traditionally been underappreciated in clinical adjustments (Elsheikh et al., 2007). Our findings suggest that flatter corneas (greater radii) systematically distort *IOPG* readings downward, leading to underestimation of true pressure. As for the elastic modulus, *ERR* grows linearly (Figures 8, 9) with increasing stiffness when other parameters are fixed. This result emphasizes the need to consider biomechanical assessments (e.g., corneal hysteresis or elastography data) when interpreting tonometric readings. In stiffer corneas—such as those affected by keratoconus treatment, aging, or prior refractive surgery—the overestimation of *IOPG* may be clinically significant. These functional relationships were derived using realistic reference values (*e.g., CCT = 0.536 mm, E = 0.16 MPa, curvature radius = 7.15 mm*), and the model outputs (Figure 10) match well with known physiological ranges. When the corneal parameters match these reference values, the *IOPG* converges closely with *IOPT*, validating the model’s internal consistency. Moreover, graphical analyses illustrate how *ERR* behaves under multiple corneal conditions. Notably, (Figure 9), provides a multi-variable visualization of the combined effect of all three parameters on measurement error, which could serve as the basis for lookup tables or software correction tools in clinical settings. The present work provides analytical and simulation-based evaluation of the theoretical model. A complementary prospective clinical study applying the dual-applanation prism *in vivo*, including comparison with dynamic contour tonometry and full statistical analysis, has been submitted separately and is intended to provide experimental validation of the theoretical framework presented here. The clinical implication is a physics-based correction method that can be applied retroactively to *IOPG* readings, enhancing measurement precision without altering the tonometer itself. The model could be implemented via software interfaces or integrated into diagnostic platforms that already measure corneal biomechanics. Future directions include *in vivo* validation, integration with real-time corneal imaging modalities such as Scheimpflug or OCT elastography, and possible AI-based refinement to predict IOPT from individualized biomechanical inputs. Ultimately, this work represents a step toward personalized tonometry and bridges the gap between theoretical modeling and clinical applicability. The model performance may vary in eyes with altered biomechanics (e.g., post-refractive surgery, keratoconus). Since the model explicitly includes corneal thickness and curvature, it can be adapted using patient-specific parameters. However, in cases of significantly altered biomechanics, further validation is required. Compared with previous literature, our model not only confirms the influence of *CCT* and elasticity (Orssengo and Pye, 1999; Elsheikh et al., 2006), but also quantifies the effect of curvature more precisely, in agreement with theoretical implications derived by Elsheikh et al. and others (Elsheikh et al., 2007). This highlights the novelty and clinical relevance of including curvature explicitly in *IOP* correction algorithms. The acquisition of two independent applanation values (*IOPG*_*0*_ *and IOPG*_*1*_) was enabled by a custom-designed dual-zone applanation cone, allowing real-time data input for the model without disrupting standard clinical procedures. A growing body of literature has highlighted the complex interplay between corneal biomechanics, structural organization, and global ocular rigidity in determining the accuracy of tonometric measurements, as well as the importance of integrating biomechanical and tomographic data for improved clinical interpretation of intraocular pressure (Luce, 2005; Roberts, 2000; Roberts, 2014; Brazuna et al., 2023; Ambrosio et al., 2017; Meek and Knupp, 2015; Pallikaris et al., 2005). In conclusion, the mathematical outputs of the model validate its conceptual structure and offer a path toward objective correction of *IOPG* based on measurable patient-specific corneal data. Future applications may include dynamic integration into clinical devices, thereby improving diagnostic precision for glaucoma management. Each of these assumptions may influence the mechanical response of the cornea and consequently the estimated IOP, particularly under non-physiological or highly variable biomechanical conditions. Their potential impact should therefore be considered when interpreting model outputs in clinical practice.

## Limitations

a. Linear elasticity
  – The model assumes linear elasticity, whereas corneal tissue exhibits nonlinear and viscoelastic behavior.

b. Isotropy
  – The isotropic approximation neglects anisotropic collagen architecture

c. Rigid fixation
  – Rigid limbal fixation may overestimate physiological constraint

d. Tear film variability
  – Surface tension was assumed constant, although spatial and temporal variability may occur

e. Clinical practicality
  – Clinical feasibility and reproducibility of dual-zone applanation require experimental confirmation

f. Sensitivity analysis
  – A formal sensitivity analysis was not included in this initial theoretical investigation and represents an important direction for future research.

From a public health perspective, a physics-based correction method applicable to standard tonometry could improve access to more accurate IOP assessment without the need for expensive or complex instrumentation. This may contribute to improved glaucoma screening and management, particularly in low-resource settings.

## Conclusion

This study presents a comprehensive physical and mathematical framework for modeling the forces involved in corneal applanation, with the explicit aim of improving the accuracy of intraocular pressure (*IOP*) measurements obtained by Goldmann applanation tonometry (*GAT*). By analytically defining the roles of intraocular thrust, corneal elasticity, tear film adhesion, and applied force, the proposed model provides a transparent and physiologically grounded method for estimating true *IOP* (*IOPT*). Unlike previous approaches, this model integrates individualized biomechanical parameters—central corneal thickness, curvature radius, and elastic modulus enabling a personalized correction of *GAT*-derived pressure values. The theoretical simulations demonstrate that the error between *GAT* and true pressure (*ERR*) is systematically influenced by these variables, offering not only mechanistic insights but also a clinically actionable correction algorithm. Further *in vivo* validation and clinical trials are warranted to confirm these findings and assess the model’s implementation in real-world diagnostic workflows. The model’s clinical feasibility is supported by an optical modification of the applanation interface, enabling practical application of the proposed equations.

## Data Availability

The raw data supporting the conclusions of this article will be made available by the authors, without undue reservation.

## Glossary

**IOPT**: True Intra-Ocular Pressure
**IOPG**: Goldmann Intra-Ocular Pressure
**F1**: force due to true intraocular pressure
**F2**: elastic reaction force of the applanated cornea
**F3**: force of attraction due to the tear film
**F4**: thrust force of the tonometer head against the applanated cornea
**h**: apical corneal thickness
**a**: average apical corneal curvature radius
**g**: applanated corneal surface radius
**r**: radial coordinate of the applanated corneal surface
**D**: Flexural bending rigidity of the corneal shell
**z**: vertical coordinate of the corneal surface to be applanated
**z0**: maximum vertical displacement of the corneal surface to be applanated
**w**: radial displacement of the corneal surface to be applanated
**v**: tangential displacement of the corneal surface to be applanated
**p**: force directed in a radial direction per unit of length
**pr**: force directed tangentially per unit of length
**R**: radius of curvature of the meniscus of the tear film
**a0**: distance between the applanation surface and the center of apical corneal curvature
**Nφ**: direct tension in the direction of the latitudinal polar coordinate
**Nθ**: direct tension in the direction of the longitudinal polar coordinate
**Nr**: direct tension in radial direction
**Mφ**: bending moment in the direction of the latitudinal polar coordinate
**Mθ**: bending moment in the direction of the longitudinal polar coordinate
**Mr**: bending moment in radial direction
**Qφ**: shear stress along the direction of the latitudinal polar coordinate
**Qθ**: shear stress along the direction of the longitudinal polar coordinate
**Qr**: shear stress in the radial direction
**φ**: latitudinal polar coordinate
**θ**: longitudinal polar coordinate
**r0**: radius of curvature along the longitudinal polar coordinate
**r1, r2**: radius of curvature along the latitudinal polar coordinate
**Y**: force per unit area along the longitudinal polar coordinate
**Z**: force per unit area in orthogonal sense
**ν**: Poisson’s ratio
**E**: modulus of elasticity (Young’s modulus)
E0: modulus of elasticity (Young’s modulus) of a standard cornea
**h0**: standard apical corneal thickness
**λ**: surface tension of the tear film
**Δp**: Laplace pressure difference
**ERR**: error of the Goldmann tonometer

## References

[B1] Ambrosio R. Jr Lopes B. T. Faria-Correia F. Salomão M. Q. Bühren J. Roberts C. J. (2017). Integration of scheimpflug-based corneal tomography and biomechanical assessments for enhancing ectasia detection. J. Refract Surg. 33 (7), 434–443. 10.3928/1081597X-20170426-02 28681902

[B2] Brazuna R. Alonso R. S. Salomão M. Q. Fernandes B. F. Ambrósio R. Jr (2023). Ocular biomechanics and glaucoma. Vis. (Basel) 7 (2), 36. 10.3390/vision7020036 37218954 PMC10204549

[B3] Cerrano E. (1909). Ricerche fisico-chimiche sulle lacrime in relazione alla pratica dei collirii, Archivio di Farm. Sper Roma, 347–358.

[B4] Chihara E. (2008). Assessment of true intraocular pressure: the gap between theory and practical data. Surv. Ophthalmol. 53 (3), 203–218. 10.1016/j.survophthal.2008.02.005 18501267

[B5] Elsheikh A. Wang D. Kotecha A. Brown M. Garway-Heath D. (2006). Evaluation of goldmann applanation tonometry using a nonlinear finite element ocular model. Ann. Biomed. Eng. 34 (10), 1628–1640. 10.1007/s10439-006-9191-8 17006754

[B6] Elsheikh A. Wang D. Brown M. Rama P. Campanelli M. Pye D. (2007). Assessment of corneal biomechanical properties and their variation with age. Curr. Eye Res. 32 (1), 11–19. 10.1080/02713680601077145 17364730

[B7] Goldmann H. Schmidt T. (1957). Applanation tonometry. Ophthalmologica. 134 (4), 221–242. 10.1159/000303213 13484216

[B8] Liu J. Roberts C. J. (2005). Influence of corneal biomechanical properties on intraocular pressure measurement: quantitative analysis. J. Cataract. Refract Surg. 31 (1), 146–155. 10.1016/j.jcrs.2004.09.031 15721707

[B9] Luce D. A. (2005). Determining *in vivo* biomechanical properties of the cornea with an ocular response analyzer. J. Cataract. Refract Surg. 31 (1), 156–162. 10.1016/j.jcrs.2004.10.044 15721708

[B10] Meek K. M. Knupp C. (2015). Corneal structure and transparency. Prog. Retin Eye Res. 49, 1–16. 10.1016/j.preteyeres.2015.07.001 26145225 PMC4655862

[B11] Orssengo G. J. Pye D. C. (1999). Determination of the true intraocular pressure and modulus of elasticity of the human cornea *in vivo* . Bull. Math. Biol. 61 (3), 551–572. 10.1006/bulm.1999.0102 17883231

[B12] Pallikaris I. G. Kymionis G. D. Ginis H. S. Kounis G. A. Tsilimbaris M. K. (2005). Ocular rigidity in living human eyes. Invest Ophthalmol. Vis. Sci. 46 (2), 409–414. 10.1167/iovs.04-0162 15671262

[B13] Roberts C. (2000). The cornea is not a piece of plastic. J. Refract Surg. 16 (4), 407–413. 10.3928/1081-597X-20000701-03 10939720

[B14] Roberts CJ. (2014). Concepts and misconceptions in corneal biomechanics. J. Cataract Refract Surg. 40, 862–869. 10.1016/j.jcrs.2014.04.019 24857435

[B15] Schwartz N. J. Mackay R. S. Sackman J. L. (1966). A theoretical and experimental study of the mechanical behavior of the cornea with application to the measurement of intraocular pressure. Bull. Math. Biophys. 28, 585–643. 10.1007/bf02476865

[B16] Tiffany J. M. Winter N. Bliss G. (1989). Tear film stability and tear surface tension. Curr. Eye Res. 8 (5), 507–515. 10.3109/02713688909000031 2736956

[B17] Timoshenko S. Goodier J. N. (1970). Theory of elasticity. 3rd ed. McGraw-Hill.

[B18] Whitacre M. M. Stein R. (1993). Sources of error with use of Goldmann-type tonometers. Surv. Ophthalmol. 38 (1), 1–30. 10.1016/0039-6257(93)90053-a 8235993

[B19] Whitacre M. M. Stein R. A. Hassanein K. (1993). The effect of corneal thickness on applanation tonometry. Am. J. Ophthalmol. 115 (5), 592–596. 10.1016/s0002-9394(14)71455-2 8488910

[B20] Yokoi N. Maruyama K. Kinoshita S. Bron A. J. Tiffany J. M. (2003). Dynamic changes in tear meniscus curvature at the rigid contact lens edge. Cornea. 22 (3), 226–229. 10.1097/00003226-200304000-00008 12658087

