# Supplementary material for: “A dual-applanation physical model to improve accuracy in goldmann tonometry by accounting for corneal biomechanics”
Source: Front Bioeng Biotechnol. 2026 Apr 29;14:1757214. doi: 10.3389/fbioe.2026.1757214 (PMC13167997; doi:10.3389/fbioe.2026.1757214)
Supplement: Supplementary file 1 [file Supplementaryfile1.pdf]

APPENDIX A

Force (*F2*)$\frac{d}{d\phi}\left(N_{\phi} \cdot r_0\right) - N_{\theta} \cdot r_1 \cdot cos\phi - r_0 \cdot Q_{\phi} + r_0 \cdot r_1 \cdot Y = 0$

$$N_{\phi} \cdot r_0 + N_{\theta} \cdot r_1 \cdot sin\phi + \frac{d}{d\phi}\left(Q_{\phi} \cdot r_0\right) + Z \cdot r_0 \cdot r_1 = 0$$

$$\frac{d}{d\phi}\left(M_{\phi} \cdot r_0\right) - M_{\theta} \cdot r_1 \cdot cos\phi - r_0 \cdot r_1 \cdot Q_{\phi} = 0$$

The elongations of the intermediate surface and the associated bending moments can be described by the following equations [9].

$$\epsilon_{\mathrm{\phi}} = \frac{1}{\mathrm{r}_1} \cdot \frac{\mathrm{dv}}{\mathrm{d\phi}} - \frac{\mathrm{w}}{r_1}$$

$$\epsilon_{\theta} = \frac{v}{r_2} \cdot cot\,\phi - \frac{w}{r_2}$$

$$M_{\phi} = -D \cdot \left[\frac{1}{r_1} \cdot \frac{d}{d\phi}\left(\frac{v}{r_1} + \frac{d\,w}{r_1 \cdot d\,\phi}\right) + \frac{v}{r_2} \cdot \left(\frac{v}{r_1} + \frac{d\,w}{r_1 \cdot d\,\phi}\right) \cdot cot\phi\right]$$

$$M_{\theta} = -D \cdot \left[\left(\frac{v}{r_1} + \frac{d\,w}{r_1 \cdot d\,\phi}\right) \cdot \frac{cot\,\phi}{r_2} + \frac{v}{r_1} \cdot \frac{d}{d\,\phi}\left(\frac{v}{r_1} + \frac{d\,w}{r_1 \cdot d\,\phi}\right)\right]$$

Since the cornea is flattened at its apex and the smoothed area exhibits circular symmetry, the general equations can be adapted to this geometry using the notation in (Figure 1):

$$\frac{d}{dr}(N_r \cdot r) - N_{\theta} \cdot \frac{r}{a} \cdot Q_r + r \cdot p_r = 0$$

$$\frac{d}{dr}(Q_r \cdot r) + \frac{r}{a} \cdot (N_r + N_{\theta}) + r \cdot p = 0$$

$$\frac{d}{dr}(M_r \cdot r) - M_{\theta} - r \cdot Q_r = 0$$

$$\varepsilon_r = \frac{1}{E \cdot h} \cdot (N_r - \nu \cdot N_{\theta}) = \frac{dv}{dr} - \frac{w}{a}$$

$$\varepsilon_{\theta} = \frac{1}{E \cdot h} \cdot (N_{\theta} - \nu \cdot N_r) = \frac{v}{r} - \frac{w}{a}$$

$$M_r = -D \cdot (\chi_r + \nu \cdot \chi_{\theta}) = -D \cdot \left(\frac{d^2w}{d\,r^2} + \frac{\nu}{r} \cdot \frac{d\,w}{dr}\right)$$

$$M_{\theta} = -D \cdot (\chi_{\theta} + \nu \cdot \chi_r) = -D \cdot \left(\frac{1}{r} \cdot \frac{dw}{dr} + \nu \cdot \frac{d^2\,w}{dr^2}\right)$$

Bending stiffness (flexural rigidity) of the cornea modeled as a thin spherical shell defined as:

$$D = \frac{E \cdot h^3}{12 \cdot (1 - \nu^2)}$$

The elastic reaction force exerted by the smoothed area of the cornea against the tonometer cone is non-uniform. It is distributed according to the following function:

$$\frac{\mathrm{E} \cdot \mathrm{h} \cdot 2\mathrm{w}}{\mathrm{a}^2 \cdot (1 - \mathrm{v}^2)} + \frac{\mathrm{D}}{\mathrm{r}} \cdot \left[\frac{\mathrm{d}^3\mathrm{w}}{\mathrm{d\,r}^3} + \frac{\mathrm{d}}{\mathrm{dr}}\left(\frac{1}{\mathrm{r}}\frac{\mathrm{d\,w}}{\mathrm{dr}}\right)\right] - \frac{2\mathrm{D}}{\mathrm{r}} \cdot \left[\frac{\mathrm{d}^3\mathrm{w}}{\mathrm{d\,r}^3} + \frac{\mathrm{d}}{\mathrm{dr}}\left(\frac{1}{\mathrm{r}}\frac{\mathrm{d\,w}}{\mathrm{dr}}\right)\right] - \mathrm{D}\left[\frac{\mathrm{d}^4\mathrm{w}}{\mathrm{d\,r}^4} + \frac{\mathrm{d}^2}{\mathrm{dr}^2}\left(\frac{1}{\mathrm{r}}\frac{\mathrm{d\,w}}{\mathrm{dr}}\right)\right]$$

From the above relationship, we can infer that the total force exerted by the elastic reaction over the smoothed area—normalized by the applanated surface can be approximated as follows, excluding higher-order terms:

$$\frac{Ehg^2}{2(1-\nu)a^3}$$

The value of the Poisson's coefficient *v* of a cornea is equal to *0.49*.

The elastic deformation of the cornea can be calculated simply by assuming that each portion of corneal tissue moves in a direction perpendicular to the surface of the cone during the smoothing operation [13].

Corneal Modulus of elasticity

The modulus of elasticity *E* of a standard cornea (*E0*) can be obtained by equating the difference in mean pressure (referred to the smoothed corneal surface) caused by the tear film, to the pressure $\frac{F2}{\pi g^2}$ due to the elastic reaction of the cornea following the applanation:

$$\frac{E_0 \cdot h_0 \cdot g^2}{2(1-v)a^3} = \frac{F3}{\pi g^2}$$

g = 1.53 mm is the radius of the smoothed corneal surface; *h0 = 0.536 mm* is the standard apical corneal thickness;

a = 7.15 mm is the mean apical curvature radius of the corneal surface;

v = 0.5 is the Poisson's ratio, which is practically equal to ½ for a cornea soaked in water.

If the difference in mean pressure (referred to the smoothed corneal surface) is estimated using the surface tension value of the distilled water as the surface tension value of the tear film, we have:

$$\frac{E_0 \cdot h_0 \cdot g^2}{2(1-v)a^3} \approx 6.6\ mm\ Hg$$

From this formula, it can be argued that the modulus of elasticity *E* of a standard cornea is approximately equal to:

$$E_0 \approx 0.26\ MPa$$

If the surface tension of the tear film is approximately *0.050 N / m2*, as indicated by other studies [10,11], then the modulus of elasticity *E0* of a standard cornea is approximately equal to:

$E_0 \approx 0.18\ MPa$

If the surface tension of the tear film of a normal eye is approximately *0.044 N / m2,* as reported in another study [11], then the modulus of elasticity *E0* of a standard cornea is approximately equal to:

$$E_0 \approx 0.16\ MPa$$

If the surface tension value of the tear film is that calculated for a normal eye, then the difference in mean pressure (on the applanated corneal area) caused by the tear film is approximatively *4.0 mmHg* and the *E0* modulus of elasticity of a standard cornea is about *0.16MPa*.
