# Supplementary material for: “A dual-applanation physical model to improve accuracy in goldmann tonometry by accounting for corneal biomechanics”
Source: Front Bioeng Biotechnol. 2026 Apr 29;14:1757214. doi: 10.3389/fbioe.2026.1757214 (PMC13167997; doi:10.3389/fbioe.2026.1757214)
Supplement: Supplementary file 2 [file Supplementaryfile2.pdf]

APPENDIX B

Force (*F3*)

$$\Delta p = \lambda \cdot \left(\frac{1}{R} - \frac{1}{\overline{BH}}\right)$$

Given that the radius of curvature *R* of the meniscus is significantly smaller than the in-plane curvature radius, the surface tension component simplifies to

$$\Delta p \cong \lambda \cdot \left(\frac{1}{R}\right)$$

λ = surface tension of the tear film

If the surface tension of the tear film is approximated to that of water: ($\lambda H2O = 0.073\ N/m$) According to Cerrano[11], the surface tension of the tear film is: ($\lambda = 0.05\ N/m$).

According to Tiffany et al. [8], the surface tension of the tear film is approximately *0.044 N/m* in normal eyes and *0.050 N/m* in dry eyes: (*λ normal = 0.044 N/m)* ; λ (*dry = 0.050 N/m*). The pressure difference across the air-liquid interface is computed by substituting the meniscus radius *R* into the above expression. This value is negative, representing the attractive force between the cone and the cornea. The direct calculation of the radius *R* is complex and depends on pre-applanation tear film thickness. However, (*F3*) can be approximated without directly calculating *R* by multiplying the Laplace pressure difference with the wetted contact surface:

$$F3 = 2\pi \cdot \overline{AB} \cdot \left(\overline{BH} + \frac{\overline{AB}}{2}\right) \cdot \Delta p = 2\pi \cdot \overline{AB} \cdot \left(\overline{BH} + \frac{\overline{AB}}{2}\right) \cdot \lambda \cdot \left(\frac{1}{R}\right)$$

Thus, the average pressure exerted by the tear film on the applanated corneal surface becomes:

$$2\pi \cdot \overline{AB} \cdot \frac{\overline{BH} + \frac{\overline{AB}}{2}}{\pi \cdot \overline{BH}^2} \cdot \Delta p = 2 \cdot \overline{AB} \cdot \frac{\overline{BH} + \frac{\overline{AB}}{2}}{\overline{BH}^2} \cdot \lambda \cdot \left(\frac{1}{R}\right)$$

This expression can be simplified by noting that the thickness ($\overline{AB}$) of the tear ring is small compared to the radius ($\overline{BH}$) of the flattened area, as depicted in (Figure 11):

$$2 \cdot \overline{AB} \cdot \frac{\overline{BH} + \frac{\overline{AB}}{2}}{\overline{BH}^2} \cdot \lambda \cdot \left(\frac{1}{R}\right) \cong 2 \cdot \overline{AB} \cdot \frac{1}{\overline{BH}} \cdot \lambda \cdot \left(\frac{1}{R}\right)$$

Making the assumption that the contact angle between the tear meniscus and the cone surface is zero and considering that angle *BOC* is small, angle *ABC* closely approximates angle *BOH*. Additionally, segments *AD* and *CD* are equal, being equal to the radius *R*. As a result, the ratio *R* / (*AB*) is given by:

$$\frac{R}{\overline{AB}} = tan\left(\frac{ABC}{2}\right) \cong tan\left(\frac{BOH}{2}\right) = \frac{sin(BOH)}{1 + cos(BOH)} = \frac{\overline{BH}}{a + a_0}$$

Hence, the average pressure difference caused by the tear film on the smoothed corneal surface (πg^2) is approximately:

$$2 \cdot \overline{AB} \cdot \frac{1}{\overline{BH}} \cdot \lambda \cdot \left(\frac{1}{R}\right) \cong 2 \cdot \frac{a + a_0}{\overline{BH}^2} \cdot \lambda$$

and the corresponding force (*F3*) is approximately:

$F3 = 2\pi \cdot \overline{AB} \cdot \left(\overline{BH} + \frac{\overline{AB}}{2}\right) \cdot \lambda \cdot \left(\frac{1}{R}\right) \cong 2\pi\lambda \cdot (a + a_0) = 2\pi\lambda \cdot \left(a + \sqrt{a^2 - g^2}\right)$The radial displacement "*w*" and tangential displacement *v* are 2 forces to consider fitting all the mathematical equations dominating the applanation. For symmetry reasons, the tangential displacement *v* is zero at the centerline (*θ=0*). Several assumptions can be made about *v*: the cornea moves freely along the smoothing surface without being restrained by the contact with the cone and that it moves in a radial direction towards the center of curvature of the cornea; the cornea adheres to the cone as it is pushed so there is no horizontal displacement; an intermediate situation between the two previous ones. In any case, the corneal displacement in a tangential direction must be considered negligible along the applanated area. In fact, this area is so small that the difference between the applanated corneal area arch and the chord underlying it is about 0.6% of the length of the arch and less than 0.8% of the diameter of the applanated area. We can simplify that *v = 0*, which is equivalent to considering that the corneal deformation during the *GAT* is a movement only towards the center of curvature of the cornea in every corneal part.

Calculation of the theoretical maximum lateral displacement

The theoretical maximum lateral displacement of the cornea with respect to the cone during applanation can be calculated by assuming that the cornea adheres to the cone so that each portion moves only perpendicular to the cone applanating area. The displacement*v* in the tangential direction is given by the product of the vertical displacement *z* with the ratio between the radial distance *r* and the corneal radius of curvature a:

$$v = z \cdot \frac{r}{a} = \left(\sqrt{(a^2 - r^2)} - \sqrt{(a^2 - g^2)}\right) \cdot \frac{r}{a}$$

The radial displacement *w* is therefore equal to:

$$w = z \cdot \frac{\sqrt{(a^2 - r^2)}}{a}$$

It can be deduced that the displacement *w* in the radial direction is a multiple of the displacement*v* in the tangential direction by a factor *a / r,* which is at least equal to *4.67.* Consequently, the displacement in the tangential direction v can be neglected as it is small compared to the displacement in the radial direction "w".
